# Supplementary material for: The Role of GPR109a Signaling in Niacin Induced Effects on Fed and Fasted Hepatic Metabolism
Source: Int J Mol Sci. 2021 Apr 13;22(8):4001. doi: 10.3390/ijms22084001 (PMC8069761; doi:10.3390/ijms22084001)
Supplement: Supplementary file 1 [file ijms-22-04001-s001.pdf]

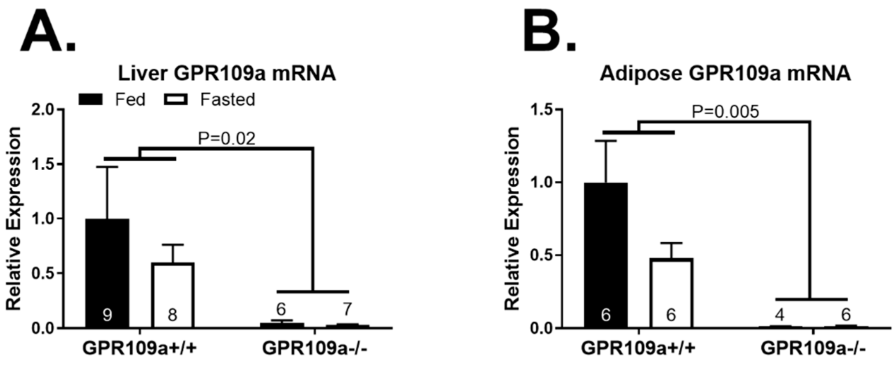

Supplemental Figure 1. Validation of GPR109a knockout. GPR109a mRNA expression in (A) liver and (B) adipose tissue. The effect of genotype was assessed by mixed model ANOVA. Number inside or above bar denotes n per group.
